# A Novel Bruton’s Tyrosine Kinase Inhibitor Suppresses Pancreatic Neuroendocrine Neoplasms Progression via ATF3-Induced Ferroptosis

**DOI:** 10.3390/cancers18142277

**Published:** 2026-07-15

**Authors:** Ping Hu, Lijun Yan, Bingyan Xue, Na He, Jianqiang Qian, Xintong Lu, Min Liu, Yanling Xu, Xu Han, Mujie Ye, Qiyun Tang

**Affiliations:** 1 Department of Neuroendocrine Tumor, the First Affiliated Hospital with Nanjing Medical University, Neuroendocrine Tumor Diagnosis and Treatment Center of Jiangsu Province, Institute of Neuroendocrine Tumor of Nanjing Medical University, Nanjing 210029, China; 2 Department of Geriatric Gastroenterology, the First Affiliated Hospital with Nanjing Medical University, Nanjing 210029, China; 3 School of Traditional Chinese Pharmacy, China Pharmaceutical University, Nanjing 211100, China; 4 Department of General Practice, the First Affiliated Hospital with Nanjing Medical University, Nanjing 210029, China

**Keywords:** pancreatic neuroendocrine neoplasms, ferroptosis, Bruton’s tyrosine kinase inhibitors, activating transcription factor 3

## Abstract

**Objective:** Current therapeutic regimens for pancreatic neuroendocrine neoplasms (pNENs) remain limited and fail to yield notable improvements in overall survival. Therefore, the development of novel agents is of paramount importance. Bruton’s tyrosine kinase inhibitors (BTKis) have demonstrated promising therapeutic potential in solid tumors; however, ibrutinib, a classic BTKi, exhibits unsatisfactory clinical efficacy against pNENs. In this study, we synthesized a novel pyrrolopyrimidine-based BTKi, QY21, and aimed to investigate its inhibitory effects on pNEN cell proliferation both in vitro and vivo and identify the core signaling pathways mediating its suppressive effects on pNENs. **Methods:** CCK-8, EdU, and colony formation assays were conducted to assess the effect of QY21 on pNENs in vitro. Transcriptome sequencing, quantitative real-time PCR, Western blotting, and flow cytometry were employed to explore the mechanisms. A xenograft tumor model in nude mice was established for in vivo validation. **Results:** QY21 significantly suppressed pNENs proliferation in vitro. Compared with the control and ibrutinib groups, QY21 exhibited stronger tumor growth inhibition in vivo. Histopathological analysis revealed a decreased Ki-67 index in the QY21 group, with no significant organ-toxic lesions observed. Transcriptome sequencing identified ATF3 as the core mediator responsible for the anti-proliferative effect of QY21. ATF3 was poorly expressed in pNENs, while QY21 markedly upregulated ATF3 expression. Mechanistically, QY21 induced ferroptosis by elevating ATF3 levels. The knockdown of ATF3 or administration of ferrostatin-1 significantly attenuated the anti-proliferative capacity of QY21, accompanied by reduced accumulation of reactive oxygen species and lipid peroxidation. **Conclusions:** This study demonstrates that the novel BTKi QY21 suppresses pNENs proliferation by triggering ATF3-mediated ferroptosis, providing a potential preclinical strategy for pNENs.

## Simple Summary

Therapeutic options for pancreatic neuroendocrine neoplasms (pNENs) remain limited, making the development of novel agents of great importance. Bruton’s tyrosine kinase inhibitors (BTKis) have shown promising therapeutic potential in solid tumors; however, ibrutinib demonstrates poor efficacy against neuroendocrine neoplasms. In this study, we synthesized a novel pyrrolopyrimidine-based BTKi, QY21, and demonstrated that QY21 significantly suppressed the proliferation of pNENs both in vitro and in vivo. Mechanistically, QY21 markedly upregulated ATF3 expression in pNENs, inducing the accumulation of reactive oxygen species and lipid peroxidation. Genetic knockdown of ATF3 or administration of the ferroptosis inhibitor ferrostatin-1 significantly attenuated the anti-proliferative capacity of QY21. In summary, our study characterized a novel BTKi, QY21, which suppresses pNENs proliferation by triggering ATF3-mediated ferroptosis. These findings may provide a potential therapeutic strategy for pNENs.

## 1. Introduction

Pancreatic neuroendocrine neoplasms (pNENs), which account for approximately 10% of all gastroenteropancreatic neuroendocrine neoplasms (GEP-NENs), were historically regarded as rare diseases with a global incidence ranging from 0.11 to 0.94 per 100,000 population [1,2]. However, pNENs have received more attention with a gradually increasing incidence in the past decades. According to the data of Surveillance, Epidemiology, and End Results, the incidence of pNENs in the United States had reached 1.31 per 100,000 in 2021 with a 20-year limited-duration prevalence of 0.025–0.03% [3]. It not only imposes physical and psychological suffering on individual patients, such as pain, anxiety and diarrhea, but also places a substantial burden on medical resources and socioeconomic systems [4,5]. Previous studies indicated that neuroendocrine neoplasms (NENs) in the pancreas had the poorest prognosis among all the GEP-NENs, with the shortest median overall survival (OS) of about 5.5 to 7 years and the 5-year cancer-specific survival was about 71.7% [3,6,7]. At initial diagnosis, up to 42–60% of patients already present with distant metastases and lose the opportunity for curative surgery [6,8,9]. The median OS for the pNENs patients with distant metastasis was only 24 months, and the 5-year survival rate was only 28.6% [6]. For unresectable advanced pNENs, systemic drug therapy, including long-acting somatostatin analogues, targeted therapy such as sunitinib and surufatinib, and cytotoxic therapy, are recommended by the current guidelines [10,11,12,13,14]. However, long-term follow-up results show that although these drugs have prolonged progression-free survival (PFS) to varying degrees [15,16,17,18], they have no significant improvement in OS [16,19,20,21]. Meanwhile, the side effects such as bleeding, hypertension, and proteinuria brought by existing targeted drugs in clinical practice cannot be ignored. Furthermore, the emergence of acquired resistance further limits their application. Therefore, the exploration of novel targeted therapeutic agents with high efficacy and low toxicity is of great importance for improving the long-term prognosis of pNENs.

Unlike classic apoptotic and necrotic death patterns, ferroptosis constitutes an independent type of regulated cell demise. Its core hallmark lies in iron-catalyzed lethal lipid peroxidation, a metabolic disturbance that drives fatal membrane injury. In recent years, it has attracted extensive attention in the field of oncology. Ferroptosis can not only directly inhibit tumor cell proliferation, but also enhance immunotherapeutic responses, reverse tumor drug resistance, and ameliorate radioresistance [22,23,24]. Activating transcription factor 3 (ATF3) belongs to the ATF/cAMP response element-binding protein superfamily. Typically, ATF3 is expressed at a low level in normal and quiescent cells, and is significantly upregulated upon exposure to stimuli such as toxicants, cytokines and oxidative stress. Accumulating evidence indicates that ATF3 serves as a central stress-responsive factor in tumor cells, which profoundly regulates glucose, amino acid and lipid metabolism. Low ATF3 expression generally predicts unfavorable clinical outcomes across most malignancies [25,26,27]. Overexpression of ATF3 was related to downregulation of Cyclin D1, blockade of cell cycle and increase in apoptosis in HepG2 cells, thereby inducing ferroptosis through multiple pathways and suppressing tumor progression [25,28,29]. Dazhi Fu et al. found that ATF3 could also promote ferroptosis by inhibiting the Nrf2/Keap1/xCT antioxidant signaling pathway and reverse cisplatin resistance in gastric cancer [30]. However, ATF3 plays a dual role in colorectal cancer. Overexpression of ATF3 inhibited migration and invasion in HCT116 cells and promoted TNF-related apoptosis [25]. Whereas, Junjia Liu et al. suggested that activation of the ATF3-CBS axis assisted SW480 cells in resisting ferroptosis and promoted colorectal cancer progression [31]. The relationship between ATF3 and ferroptosis in pancreatic neuroendocrine neoplasms remains unelucidated.

Bruton’s tyrosine kinase (BTK) is a member of non-receptor tyrosine kinases belonging to the Tec family, which is widely expressed in most hematopoietic cells except T cells and plasma cells. Bruton’s tyrosine kinase (BTK) consists of five conserved functional domains, including the N-terminal pleckstrin homology (PH) domain, a Tec homology (TH) domain, tandem Src homology 3 (SH3) and Src homology 2 (SH2) domains, and the C-terminal kinase domain (SH1). The PH domain recognizes phosphatidylinositol 3,4,5-trisphosphate (PIP_3_) to recruit BTK to the plasma membrane upon BCR stimulation. A TH domain, followed by tandem SH3 and SH2 domains, mediates intermolecular protein–protein interactions to assemble downstream signal complexes. The C-terminal tyrosine kinase domain harbors the ATP-binding pocket and catalytic active site, enabling autophosphorylation and phosphorylation of downstream substrates, thus regulating downstream signal pathways [32]. Bruton’s tyrosine kinase inhibitors (BTKis) have been approved for the treatment of chronic lymphocytic leukemia, Waldenström’s macroglobulinemia and many other hematological diseases. Furthermore, BTKis also exert anti-tumor effects in BTK-negative solid tumors by modulating various immune cells in the tumor microenvironment [33]. A previous study found that the BTK inhibitor PCI32765 could induce tumor vascular collapse and tumor regression by inhibiting mast cell degranulation in insulinoma-bearing mice [34]. Ibrutinib, the first-generation BTKi, was proved to play an enhanced role in anti-PD-L1 antibody treatment by downregulating circulating myeloid-derived suppressor cells in murine melanoma [35]. However, a phase II clinical trial in 2019 that aimed to evaluate the effect of ibrutinib on advanced NENs failed to achieve an objective response with a median PFS of only 3 months [36].

In this study, we synthesized a series of novel pyrrolopyrimidine-based BTKis and QY21 was identified as exhibiting the most potent anti-tumor activity against pNENs. Mechanistically, we found that QY21 could induce ferroptosis in pNENs by upregulating ATF3 both in vitro and in vivo. This would provide a promising therapeutic strategy for the treatment of pancreatic neuroendocrine tumors.

## 2. Materials and Methods

**Cellular cultivation:** The human pancreatic nestin-expressing ductal cell line (HPNE) was sourced from the American Type Culture Collection (CBP60857). The QGP-1 cell line derived from human pNENs was acquired from the Japanese Collection of Research Bioresources cell bank (JCRB0183). Additionally, the BON-1 cell line, also from pNENs, was provided by Prof. Xianjun Yu of the Affiliated Cancer Hospital of Fudan University. HPNE cells were cultured in Dulbecco’s Modified Eagle Medium with high glucose, supplied by Jiangsu KeyGEN Bio TECH Corp., Ltd. (Nanjing, China). QGP-1 cells were grown in RPMI-1640 media (Gibco, Grand Island, NY, USA), while BON-1 cells were maintained in DMEM/F-12 from Jiangsu KeyGEN Bio TECH Corp., Ltd. Each medium contained 10% fetal bovine serum (FBS, Yeasen, Shanghai, China) along with 1% penicillin-streptomycin. All cells were cultured in a humidified incubator at 37 °C with 5% CO_2_ and 21% O_2_, and the culture medium was refreshed every 24 h. When cells reached 80–90% confluency, they were washed twice with pre-warmed phosphate-buffered saline (PBS, pH 7.4), followed by incubation with 0.25% trypsin-0.02% EDTA solution at 37 °C for 1 min. The digestion was immediately quenched by adding two volumes of complete culture medium. The cell suspension was centrifuged at 300× *g* for 5 min at room temperature. After the supernatant was discarded, the cell pellet was resuspended in fresh complete medium and subcultured at a split ratio of 1:3. All experiments were performed with cells in the logarithmic growth phase within 20 passages after thawing.

**Construction of stably transfected cell lines:** Plasmids for ATF3 knockdown and overexpression were created using the PLKO.1 and PLVX vectors, respectively, by Beijing Tsingke Biotech Co., Ltd. (Beijing, China). Lentivirus packaging was performed in 293T cells, utilizing PEI MAX from Polysciences (Warrington, Pennsylvania, PA, USA). Afterward, the virus was harvested and concentrated for the infection of wild-type pNEN cells over a period of 48 h. Cells that were stably transfected were selected by applying treatment with 5 μg/mL puromycin (Yeasen, Shanghai, China) and confirmed through quantitative real-time polymerase chain reaction (qRT-PCR) and Western blotting (WB). The specific shRNA sequences can be found in Table S1.

**Quantitative real-time PCR (qRT-PCR):** RNA was extracted from pNEN cells utilizing a rapid extraction kit for total RNA from Nanjing Vazyme Biotech Co., Ltd. (Nanjing, China). To eliminate genomic DNA, a 5× *g* DNA digester (Yeasen, Shanghai, China) was employed. Following this, 4× Hifair^®^ III SuperMix plus (Yeasen) was incorporated for reverse transcription, enabling the synthesis of cDNA in accordance with the protocols specified by the manufacturer. The Quantstudio^TM^ 7 Flex instrument, along with a SYBR Green PCR master mix (Yeasen), was utilized for PCR following these steps: an initial predenaturation at 95 °C for 2 min, followed by 40 cycles consisting of denaturation at 95 °C for 10 s and annealing at 60 °C for 30 s; afterward, a melting curve analysis was conducted based on the standard settings of the instrument. GAPDH served as the internal reference. Data analysis was performed using the 2^−ΔΔCt^ method. The sequences of the primers can be found in Table S2.

**Western blotting (WB):** Cells were lysed in an NP-40 buffer (Beyotime Biotech Inc., Shanghai, China) supplemented with 1 mM phenylmethanesulfonyl fluoride (Beyotime, Shanghai, China) on ice for a duration of 30 min. Following this, the lysates underwent centrifugation at 12,000× *g* for 15 min at 4 °C. The resulting supernatants were collected to determine protein concentrations using a BCA protein assay kit (Beyotime). Equal protein quantities were combined with 5× SDS loading buffer (NCM Biotech, Suzhou, China), heated at 100 °C for 10 min, and then separated using a 10% SDS-PAGE gel before being transferred onto a nitrocellulose membrane. The membranes were incubated with an 8% defatted milk solution (4 g of skim milk powder in 50 mL of TBST) for 2 h at room temperature and then exposed to primary antibodies overnight at 4 °C. Afterward, the membranes were washed three times with TBST (Wuhan Servicebio Technology Co., Ltd., Wuhan, China) for 10 min each and treated with HRP-conjugated anti-rabbit or anti-mouse IgG for 1 h at room temperature, followed by three additional TBST washes. Protein bands were detected using enhanced chemiluminescence substrate (NCM Biotech, Suzhou, China) and visualized with a Tanon Gel Imaging System (Shanghai Tianneng Science and Technology Co., Ltd., Shanghai, China). All uncropped blot images with corresponding molecular weight markers, can be found in Supplementary Materials S1. Detailed information regarding the antibodies can be found in Table S3.

**Cell Counting Kit-8 (CCK-8) experiment:** QGP-1 and BON-1 cell lines were introduced into 96-well plates at concentrations of 5 × 10^3^ and 3 × 10^3^ cells per well, respectively, using 100 μL of a complete culture medium. Following the attachment of the cells, treatments with dimethylformamide (DMF) or the specified drugs were administered. At intervals of 0, 24, 48, and 72 h post-treatment, 10 μL of CCK-8 reagent (Nanjing Vazyme Biotech Co., Ltd., Nanjing, China) was added to each well, and the plates were then incubated for 2 h at 37 °C within an incubator. Subsequently, absorbance was recorded at 450 nm utilizing a microplate reader.

**Colony formation assay:** A total of 1000 QGP-1 or BON-1 cells were individually plated in 6-well plates and incubated overnight. Subsequently, either DMF or the specified drugs were administered. Following a 48 h treatment period, the medium containing the drugs was replaced with fresh complete medium, which was updated every 3 days. After a cultivation period of 14 days, the plates were fixed using 4% paraformaldehyde (PFA) for 15 min and then stained with 0.25% crystal violet at room temperature for 30 min. Colonies with more than 50 cells were quantified using ImageJ (Version 1.54p, National Institutes of Health, Bethesda, MD, USA).

**EdU incorporation assay:** QGP-1 and BON-1 cells were individually plated in 96-well plates at a density of 1 × 10^4^ cells per well, followed by treatment with DMF or specified drugs for a duration of 48 h once the cells had adhered. To evaluate cell proliferation, the BeyoClick™ 5-ethynyl-2′-deoxyuridine (EdU) Cell Proliferation Kit (Beyotime) was employed in accordance with the manufacturer’s guidelines. In summary, the drug-containing culture medium was substituted with fresh media supplemented with 10 µM EdU, and the cells were incubated for 2 h at 37 °C in a humidified CO_2_ environment. After this incubation, fixation was carried out using 4% PFA for 15 min at room temperature. A permeabilization step followed utilizing 0.3% Triton X-100 (BioFROXX GmbH, Einhausen, Hesse, Germany) in PBS for 15 min. Subsequently, the cells were treated with the click reaction solution for 30 min at room temperature in the absence of light, and after three washes with PBS, the cell nuclei were counterstained with Hoechst 33342 for 10 min. Images were captured using a Zeiss Axio Observer 7 inverted microscope (Carl Zeiss Microscopy GmbH, Jena, Germany) and processed with ZEN 3.0 software (blue edition) (Carl Zeiss Microscopy GmbH, Jena, Germany).

**Detection of Lipid Peroxidation and ROS levels:** Cells of BON-1 or QGP-1 were plated in 6-well plates at a density of 3 × 10^5^ cells per well and allowed to culture overnight. On the following day, fresh medium was supplemented with DMF or drugs for a 48 h treatment period in a cell incubator. After this, the drug-laden medium was discarded, and each well received 1 mL of PBS, which contained either 2 μM C11-BODIPY 581/589 (Beyotime) for evaluating lipid peroxidation or 10 μM DCFH-DA (Beyotime) for detecting ROS. Following a 20 min incubation with the probes at 37 °C, the cells were rinsed with PBS to eliminate any unbound probe. The cells were then collected and analyzed using a CytoFLEX flow cytometer (version 2.4, Beckman Coulter, Brea, CA, USA), with the data being processed through FlowJo software (version 10.9; BD Biosciences, Ashland, OR, USA).

**Immunofluorescence Staining:** Cells were placed in confocal dishes for overnight incubation to achieve 60–80% confluence. Subsequently, they underwent three rinses with PBS, followed by fixation using 4% PFA for 15 min and permeabilization with 0.5% Triton X-100 for 10 min. Afterward, the cells were blocked for 30 min at room temperature using 5% bovine serum albumin (BSA) to prevent nonspecific binding. The following day, incubation was done overnight at 4 °C with the rabbit anti-ATF3 antibody (Affinity Biosciences, Cincinnati, OH, USA). After three washes with PBST (for 5 min each), the cells were treated with Coralite488-conjugated goat anti-rabbit IgG (H+L) (Proteintech Group, Inc., Rosemont, IL, USA) for 60 min at room temperature, shielded from light. Nuclei were stained for 15 min with Hoechst 33342. Finally, after three additional washes with PBST, images were captured using Zeiss confocal fluorescence microscopy (Carl Zeiss, Germany).

**Establishment of xenograft mouse models:** To further investigate the in vivo effects of QY21 on pNENs, a nude mouse xenograft tumor model was established. A total of 24 male BALB/c nude mice, aged 4–6 weeks, were sourced from Vital River (Beijing Vital River Laboratory Animal Technology Co., Ltd., Beijing, China) and were kept in a specific pathogen-free environment at the Animal Center of Nanjing Medical University (Nanjing, China). All individual mice (each defined as one experimental unit) were randomly allocated to control and treatment groups (*n* = 4 per group), with randomisation sequences generated by SPSS (version 25, IBM Corp., Armonk, NY, USA) random case function. 5 × 10^6^ BON-1 cells were injected subcutaneously into the right axilla of the mice. The control group received 150 μL of PBS via gavage every two days starting from the seventh day after injection, whereas the treatment groups were administered either QY21 or ibrutinib (10 mg/kg) bi-daily. Cage positions were rotated periodically to eliminate positional environmental differences among cages. Tumor development and growth were observed every three days until the conclusion of the experiment or tumor volume larger than 1500 mm^3^. Toxicity was monitored throughout the experiment by recording body weight and by observing general behavior and appearance. Four weeks following the injection, all mice were euthanized, and the tumors were surgically removed and preserved in 4% PFA for subsequent analyses. Major organs (heart, liver, lung, kidney) were harvested, fixed, and subjected to histopathological examination. The tumor volume (V) was determined using the formula: V = (width^2^ × length)/2. Tumor weight was recorded using an electronic scale. All animal procedures carried out in this study were sanctioned by the Institutional Animal Care and Use Committee (IACUC) at Nanjing Medical University.

**Statistical analysis:** Data analysis was conducted using GraphPad Prism 9.0. For cellular experiments, three replicate wells were set up per group, and three independent biological samples were included for statistical comparison. For in vivo xenograft assays, all four subcutaneous tumors from each group were subjected to statistical analysis. Normality and homogeneity of variance were tested before analysis; datasets satisfying parametric assumptions were analysed via paired/unpaired Student’s *t*-test or one-way analysis of variance (ANOVA), while non-normal data were analysed using appropriate non-parametric tests. All data are presented as mean ± standard deviation (M ± SD). A *p*-value of less than 0.05 was considered to denote a statistically significant difference. Each experiment was carried out a minimum of three times.

## 3. Results

### 3.1. Synthesis of Novel Pyrrolopyrimidine-Based BTKis with Superior Anti-pNENs Activity than Ibrutinib

**Figure 1:**
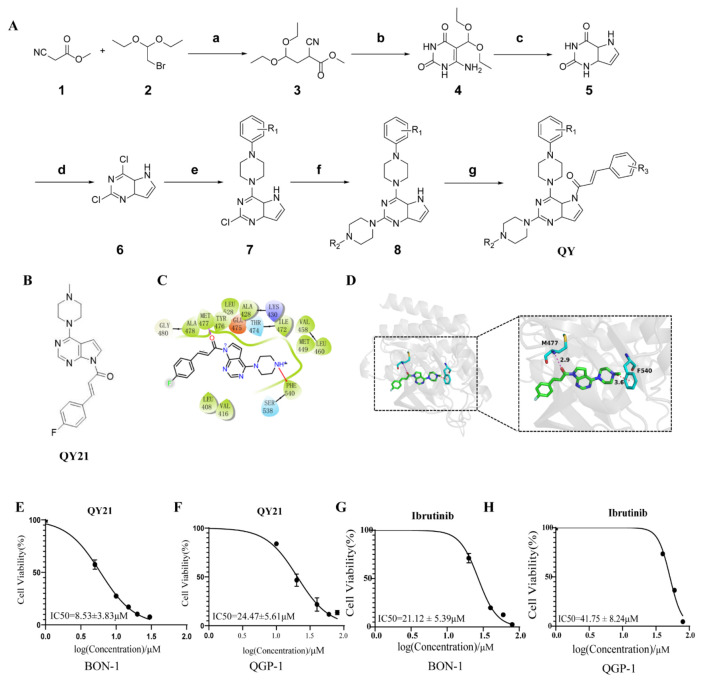
Design strategy for pyrrolopyrimidine-based BTKis. (**A**) The schematic diagram of synthetic process for novel pyrrolopyrimidine-based BTKis. (**B**) The structural formula of QY21. (**C**,**D**) Molecular docking diagram between QY21 and BTK protein. (**E**,**F**) The IC_50_ of QY21 in BON-1 and QGP-1 cells. (**G**,**H**) The IC_50_ of ibrutinib in BON-1 and QGP-1 cells.

A total of 22 kinds of pyrrolopyrimidine-based BTKis were designed through virtual library screening and synthesized via a series of chemical reactions (Figure 1A). Half-maximal inhibitory concentration (IC_50_) values of these novel BTKis in QGP-1 and BON-1 cells were determined by the CCK-8 assay. As shown in (Table 1), QY21 exhibited a lowest IC_50_ value in both QGP-1 (24.47 ± 5.61 μM) and BON-1 (8.53 ± 3.83 μM) cells among these derivatives and its structural formula was displayed in (Figure 1B). To investigate whether QY21 could directly interact with the active site of BTK, molecular docking analysis was performed. The docking results showed that QY21 could be accommodated within the active pocket of BTK, suggesting a potential direct interaction with the catalytic region of BTK (Figure 1C,D). QY21 exhibited a binding energy of −9.57 kcal/mol and an estimated inhibition constant (Ki) of 0.097 μM. The final intermolecular energy of QY21 was −9.60 kcal/mol (Table S4), indicating that protein-ligand interactions made a major contribution to the predicted binding stability. In addition, ibrutinib, a first-generation BTKi, was selected as a reference compound for comparison. Although ibrutinib showed a stronger predicted binding affinity, with a binding energy of −11.27 kcal/mol and a Ki value of 0.005 μM (Table S4), the IC_50_ of QY21 against pNENs was also lower than that of ibrutinib (Figure 1E–H). Based on the above results, QY21, which exhibited the highest activity against pNEN cells, was selected for subsequent experiments.

### 3.2. QY21 Inhibits the Proliferation of pNENs Both In Vitro and In Vivo

**Figure 2:**
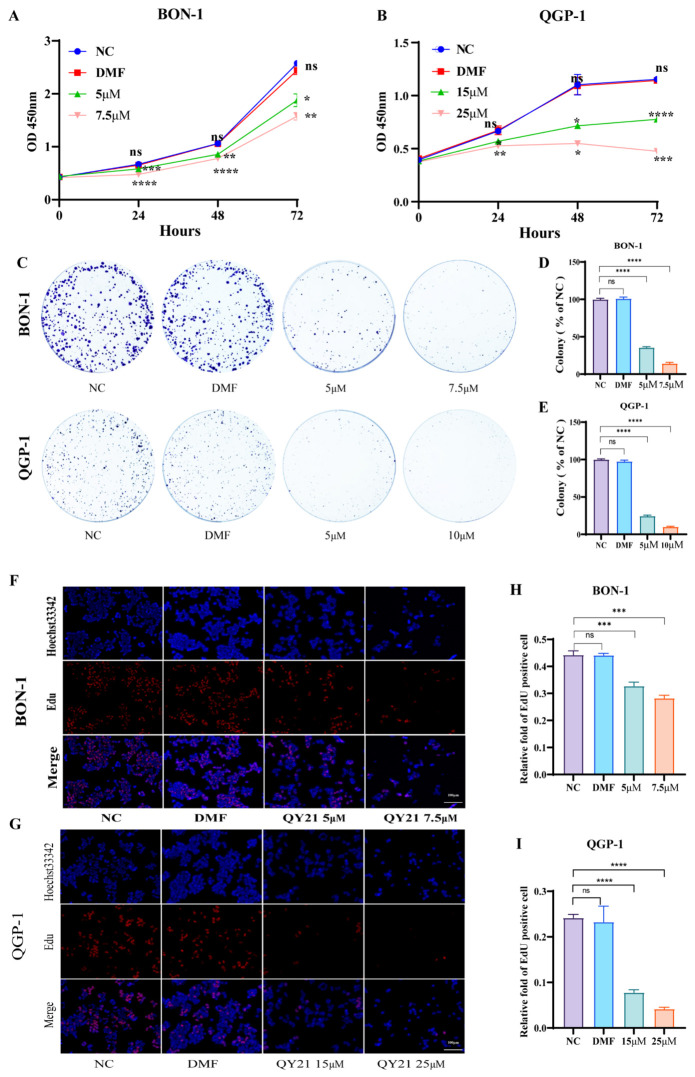
QY21 inhibited the proliferation of pNEN cells in vitro in a dose-dependent manner. (**A**,**B**) CCK-8 assay showed that QY21 inhibited the proliferation of BON-1 and QGP-1 cells (*n* = 3). (**C**–**E**) Colony formation assay indicated that QY21 impeded the proliferation of pNEN cells (Due to complete loss of colony-forming ability at 15 μM and 25 μM, QGP-1 cells were treated with 5 μM and 10 μM for colony formation assays) (*n* = 3). (**F**–**I**) EdU incorporation assay revealed that QY21 reduced the synthesis of DNA in BON-1 and QGP-1 cells (*n* = 3). Magnification: 200×. * *p* < 0.05, ** *p* < 0.01, *** *p* < 0.001, **** *p* < 0.0001.

In this study, we treated BON-1 cells with QY21 at 5 μM and 7.5 μM, while QGP-1 cells at 15 μM and 25 μM. Cell growth curves determined by CCK-8 assay demonstrated that QY21 inhibited the proliferation of BON-1 and QGP-1 cells in a dose-dependent manner, whereas, no significant difference was observed between the DMF solvent control group and the blank control group (Figure 2A,B). Furthermore, colony formation (Figure 2C–E) and EdU assays (Figure 2F–I) also verified that QY21 retarded the proliferation of pNEN cells.

**Figure 3:**
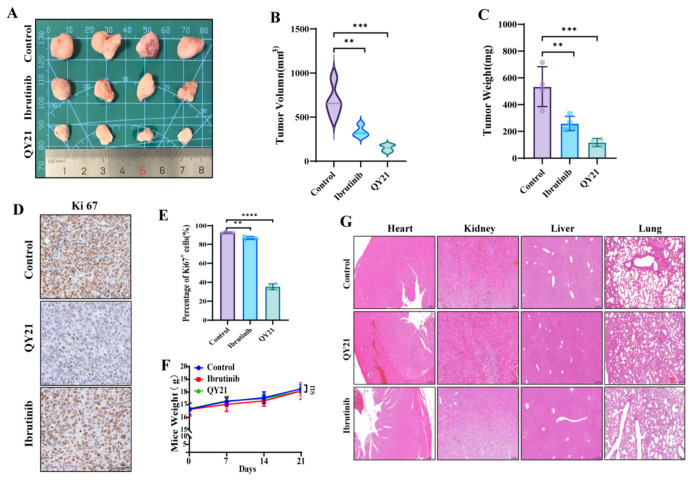
QY21 suppressed the tumor growth of pNENs in vivo. (**A**) Subcutaneous xenograft tumors derived from BON-1 cells (*n* = 4). (**B**,**C**) Tumor volume and weight in QY21 group were smaller than those in the control and ibrutinib group (*n* = 4). (**D**,**E**) Immunohistochemical staining demonstrated that tumor in the QY21 group showed the lowest Ki-67 expression among the three groups (*n* = 4). Magnification: 400×. (**F**) Mice weight in each group (*n* = 4). (**G**) Histopathological examination of major vital organs (*n* = 4). Magnification: 100×. ns: no significance, ** *p* < 0.01, *** *p* < 0.001, **** *p* < 0.0001.

A xenograft tumor model was further established in nude mice to investigate the efficacy of QY21 in vivo. The results demonstrated that both tumor volume and tumor weight were significantly reduced in the QY21 group compared with that in the control group and QY21 exerted a more potent inhibitory effect against pNENs than ibrutinib (Figure 3A–C). Immunohistochemical staining revealed that the Ki-67 index in tumors of the QY21 group was lower than that in the control and ibrutinib groups (Figure 3D,E), while no significant body weight loss (Figure 3F) or no obvious histopathological damage was observed in heart, kidney, liver, or lung of the nude mice in any group (Figure 3G). Collectively, QY21 significantly suppressed the proliferation of pNENs both in vitro and in vivo without apparent organ toxicity, indicating a favorable safety profile.

### 3.3. QY21 Upregulates Atf3 to Suppress the Progression of Pancreatic Neuroendocrine Neoplasms

**Figure 4:**
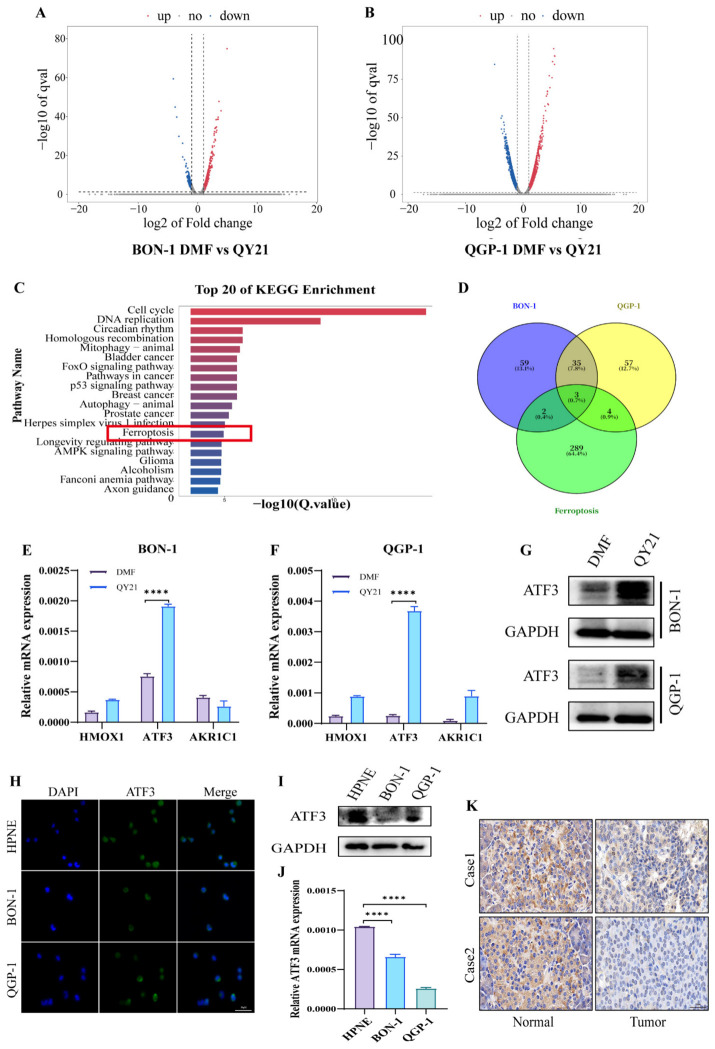
QY21 upregulated ATF3 expression in pNEN cells. (**A**,**B**) Volcano plots of DEGs in BON-1 (**A**) and QGP-1 (**B**) cells after 48 h treatment with QY21 and DMF. Red: upregulated genes; blue: downregulated genes; gray: no significant change. (**C**) KEGG analysis showed QY21 affected multiple biological processes in pNENs. (**D**) Venn diagram showed the overlap of ferroptosis-related DEGs between BON-1 and QGP-1 cells and three candidate target genes were identified. (**E**,**F**) qRT-PCR analysis verified that QY21 induced significant upregulation of ATF3 (*n* = 3). (**G**) WB demonstrated that QY21 upregulated ATF3 protein expression in BON-1 and QGP-1 cells. The uncropped blots are shown in Supplementary Materials S1. (**H**) Immunofluorescence staining of ATF3 in HPNE, BON-1, and QGP-1 cells. Magnification: 400×. (**I**,**J**) WB and qRT-PCR indicated that ATF3 expression was lower in BON-1 and QGP-1 cells than that in HPNE cells. The uncropped blots are shown in Supplementary Materials S1. (**K**) Immunohistochemical staining showed that ATF3 was downregulated in human pNENs tumor tissues than that in adjacent normal tissues. Magnification: 400×. **** *p* < 0.0001.

Transcriptome sequencing was performed to elucidate the underlying molecular mechanism by which QY21 inhibits the proliferation of pNENs. Numerous differentially expressed genes (DEGs) were identified between QY21 group and the control group in BON-1 and QGP-1 cells after 48 h treatment (Figure 4A,B). KEGG enrichment analysis revealed that these DEGs were involved in multiple critical biological processes, including ferroptosis (Figure 4C). We further intersected the upregulated DEGs in the QY21 group with ferroptosis-related genes from public databases and screened out three candidate target genes, including HMOX1, ATF3 and AKR1C1 (Figure 4D).

Quantitative real-time PCR was subsequently applied to verify the expression levels of the three genes in pNENs. The results showed that ATF3 exhibited the most significant upregulation in mRNA level in QY21-treated pNEN cells relative to the DMF group (Figure 4E,F). WB experiments further confirmed that the protein expression of ATF3 was also markedly elevated in the QY21 group both in BON-1 and QGP-1 cells (Figure 4G).

Immunofluorescence staining was conducted to investigate the basal expression of ATF3 in HPNE, BON-1 and QGP-1 cells. It was observed that ATF3 was predominantly localized in the cytoplasm and its immunofluorescence intensity was lower in pNEN cells than that in HPNE cells (Figure 4H). Consistently, the results of WB and qRT-PCR also confirmed that ATF3 expression was notably lower in pNEN cells than that in HPNE cells (Figure 4I,J). Additionally, immunohistochemical (IHC) analysis indicated that ATF3 was significantly decreased in pNENs tumor tissues compared with adjacent normal tissues (Figure 4K). In conclusion, ATF3 is lowly expressed in pNENs, and QY21 can markedly upregulate ATF3 expression in BON-1 and QGP-1 cells, which may contribute to the activation of ferroptosis in pNENs.

### 3.4. QY21 Inhibits pNENs Proliferation by Facilitating Ferroptosis, and the Suppressive Effect Is Partially Reversed by Ferrostatin-1

**Figure 5:**
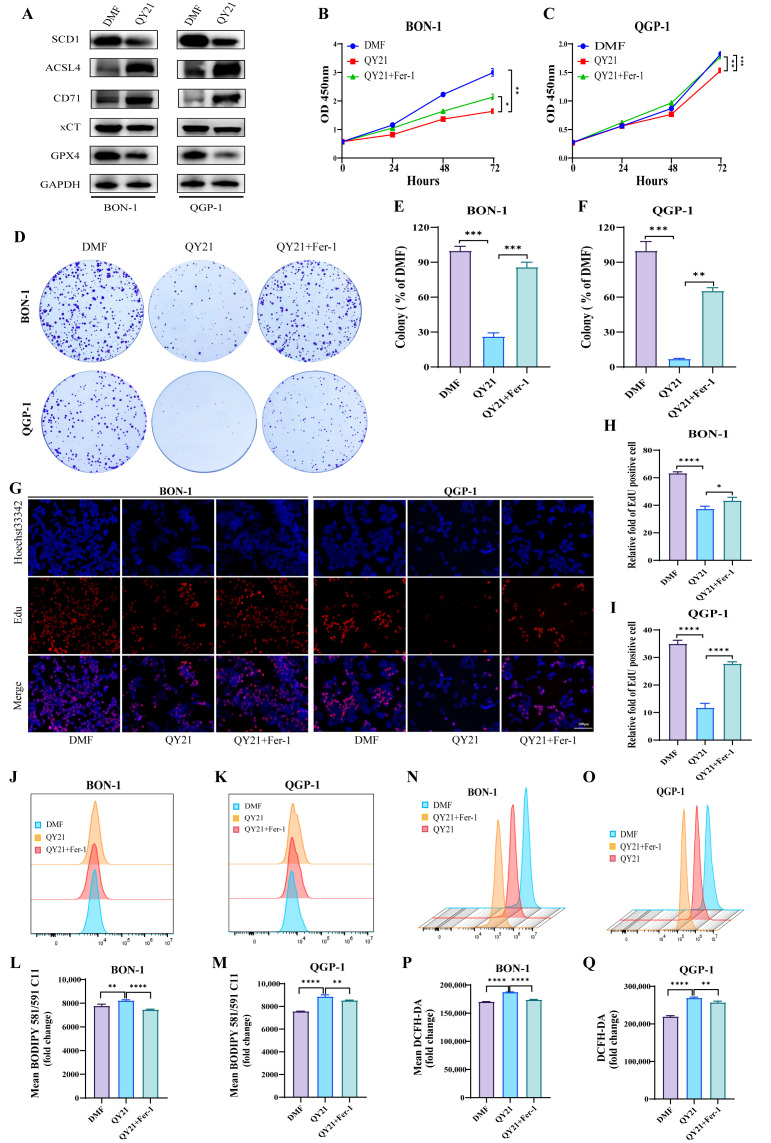
QY21 inhibited pNENs proliferation by inducing ferroptosis. (**A**) WB was performed to detect ferroptosis-related molecules. The uncropped blots are shown in Supplementary Materials S1. (**B**,**C**) CCK-8 assay showed that Fer-1 abrogated the QY21-induced loss of cell viability (*n* = 3). (**D**–**F**) Colony formation assay confirmed that Fer-1 reversed the QY21-suppressed clonogenic growth (*n* = 3). (**G**–**I**) EdU incorporation assay demonstrated that the reduction in DNA synthesis caused by QY21 was restored by Fer-1 (*n* = 3). Magnification: 200×. (**J**–**M**) Lipid peroxidation level was detected by flow cytometry and the results showed that excessive accumulation of lipid peroxidation induced by QY21 could be reversed by Fer-1 (*n* = 3). (**N**–**Q**) QY21 induced ROS accumulation and Fer-1 co-treatment could alleviate this effect (*n* = 3). * *p* < 0.05, ** *p* < 0.01, *** *p* < 0.001, **** *p* < 0.0001.

We examined some core ferroptosis-related molecules in BON-1 and QGP-1 cells by WB. The results showed that QY21 upregulated the protein levels of transferrin receptor 1 (CD71) and acyl-CoA synthetase long-chain family member 4 (ACSL4), while decreasing the expression of glutathione peroxidase 4 (GPX4), solute carrier family 7 member 11 (xCT) and stearoyl-CoA desaturase 1 (SCD1) in pNEN cells (Figure 5A), suggesting that QY21 promotes ferroptosis in pNEN cells. When QY21 was combined with ferrostatin-1 (Fer-1), a ferroptosis inhibitor, CCK-8 assay indicated that the growth rate of pNEN cells in combination group was faster than that in QY21-alone group (Figure 5B,C). Colony formation assays (Figure 5D–F) and EdU incorporation (Figure 5G–I) also confirmed that the inhibitory effect of QY21 on pNEN cell proliferation was partially reversed upon co-administration with Fer-1.

Given that ferroptosis is characterized by lipid peroxidation and ROS accumulation, flow cytometry was further performed using C11-BODIPY581/589 and DCFH-DA probes to detect lipid peroxidation and ROS levels in pNEN cells, respectively. The results showed that QY21 induced excessive accumulation of lipid peroxidation in pNEN cells, and this effect was attenuated by Fer-1 compared with QY21 alone (Figure 5J–M). Similarly, QY21 triggered prominent ROS accumulation in pNEN cells, which was partially rescued by Fer-1 co-treatment (Figure 5N–Q).

Collectively, these findings demonstrate that QY21 suppresses pNENs proliferation by inducing ferroptosis.

### 3.5. Overexpression of Atf3 Restrains the Growth of pNENs Cells and Promoted Ferroptosis

**Figure 6:**
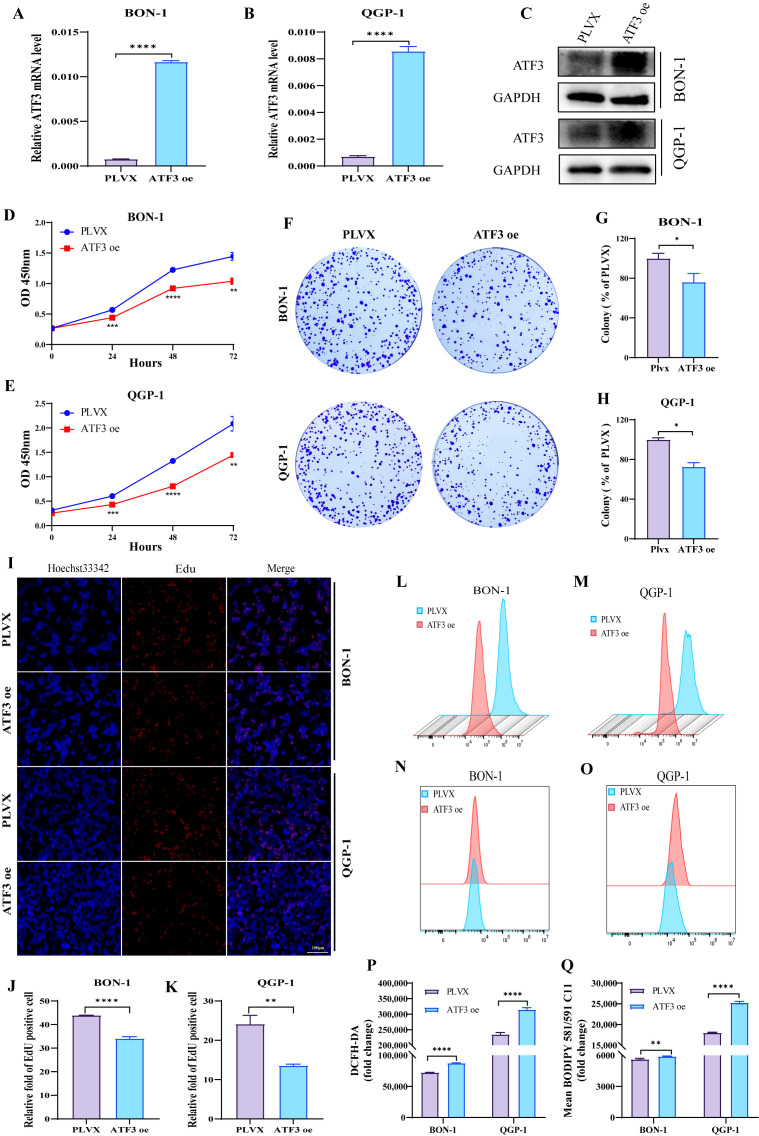
ATF3 overexpression inhibited cell proliferation and induced ferroptosis in pNENs. (**A**,**B**) qRT-PCR confirmed remarkable upregulation of ATF3 mRNA in BON-1 and QGP-1 cells (*n* = 3). (**C**) WB verified increased ATF3 protein levels in stable ATF3-overexpressing pNEN cell lines. The uncropped blots are shown in Supplementary Materials S1. (**D**,**E**) CCK-8 assays showed that ATF3 overexpression significantly reduced cell viability (*n* = 3). (**F**–**H**) Colony formation assays demonstrated impaired clonogenic capacity in ATF3-overexpression cells (*n* = 3). (**I**–**K**) EdU incorporation assays revealed a marked decrease in DNA synthesis upon ATF3 overexpression (*n* = 3). Magnification: 200×. (**L**–**Q**) Flow cytometry analysis showed that ATF3 overexpression increased lipid peroxidation and ROS accumulation (*n* = 3). * *p* < 0.05, ** *p* < 0.01, *** *p* < 0.001, **** *p* < 0.0001.

To investigate the biological function of ATF3 in pNENs, we established stable ATF3-overexpressing BON-1 and QGP-1 cell lines. The transfection efficiency was validated by qRT-PCR and WB (Figure 6A–C). CCK-8 were performed to investigate the effect of ATF3 overexpression on the proliferation of pNENs. The results revealed that ATF3 overexpression markedly impeded the proliferation of BON-1 and QGP-1 cells (Figure 6D,E). Consistent results were obtained in the colony formation assay (Figure F–H). EdU assay further confirmed that ATF3 upregulation inhibited DNA synthesis in BON-1 and QGP-1 cells (Figure 6I–K). Flow cytometry analysis demonstrated that overexpression of ATF3 significantly increased lipid peroxidation and ROS accumulation in pNENs (Figure 6L–Q). All in all, these findings indicated that ATF3 overexpression induced ferroptosis and attenuated the proliferation of pNENs.

### 3.6. Inhibition of Atf3 Suppresses Ferroptosis and Partially Reverses the Antineoplastic Effect of QY21 in pNENs

**Figure 7:**
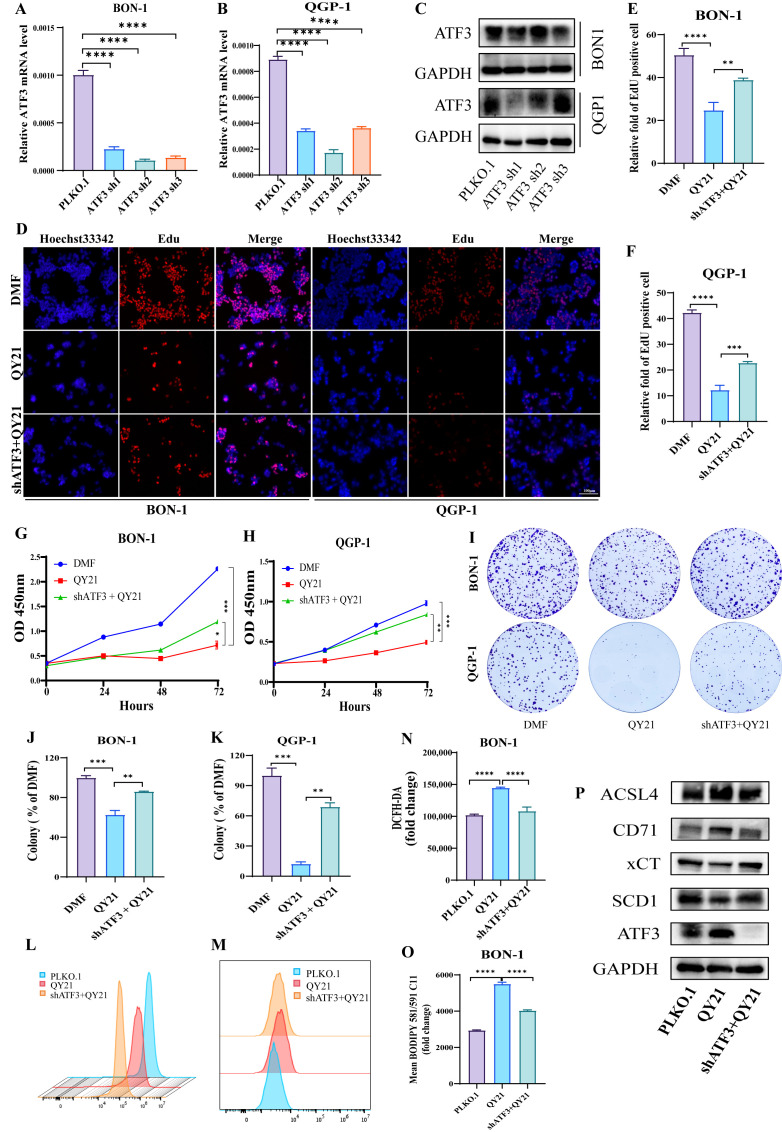
Inhibition of ATF3 suppressed ferroptosis and partially reversed the anti-neoplastic effect of QY21 in pNENs. (**A**–**C**) pNEN cells with stable ATF3 knockdown were constructed and validated by qRT-PCR and WB. The uncropped blots are shown in Supplementary Materials S1. (**D**–**F**) EdU incorporation assay showed that ATF3 knockdown partially restored QY21-induced EdU-positive cell numbers in both BON-1 and QGP-1 cells (*n* = 3). Magnification: 200×. (**G**,**H**) CCK-8 proliferation assays demonstrated that the inhibitory effect of QY21 on pNENs viability was attenuated by ATF3 knockdown (*n* = 3). (**I**–**K**) Colony formation assays confirmed that inhibition of ATF3 rescued the anti-neoplastic effect of QY21 in pNENs (*n* = 3). (**L**–**O**) QY21-induced lipid peroxidation and ROS accumulation were reduced upon ATF3 knockdown (*n* = 3). (**P**) WB analysis of ferroptosis-related proteins. The uncropped blots are shown in Supplementary Materials S1. * *p* < 0.05, ** *p* < 0.01, *** *p* < 0.001, **** *p* < 0.0001.

To clarify whether QY21 exerts its anti-tumor effects by modulating ATF3 in pNENs, we generated stable ATF3-knockdown BON-1 and QGP-1 cell lines. The knockdown efficiency was verified by qRT-PCR and WB (Figure 7A-C), and shRNA #1 with the lowest ATF3 protein expression was selected for subsequent rescue experiments. EdU assays (Figure 7D–F), CCK-8 (Figure 7G,H) and colony formation assays (Figure 7I–K) all revealed that ATF3 knockdown partially reversed the tumor-suppressive effect of QY21 on pNENs.

Flow cytometry was employed to further explore the influence of ATF3 knockdown on ferroptosis in pNEN cells. The results implied that ATF3 inhibition partially abrogated QY21-induced lipid peroxidation and ROS accumulation (Figure 7L–O). Western blotting results further demonstrated that the upregulation of CD71 and ACSL4 induced by QY21 was attenuated upon ATF3 knockdown, while the decreased expression levels of xCT and SCD1 were restored to a certain extent (Figure 7P).

**Figure 8:**
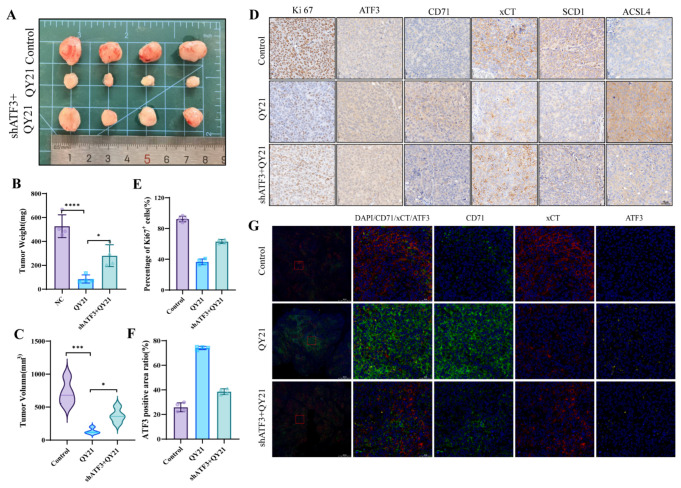
ATF3 knockdown partially abrogated the anti-tumor and ferroptosis-inducing effects of QY21 in pNEN xenografts. (**A**) Representative images of xenograft tumors (*n* = 4). (**B**,**C**) Tumor weight and volume reduced by QY21 were partially reversed when ATF3 was knocked down (*n* = 4). (**D**–**F**) The expression of Ki67, ATF3 and some ferroptosis-related markers was analyzed by IHC staining (*n* = 4). Magnification: 400×. (**G**) Representative images of immunofluorescence co-staining for DAPI (nucleus, blue), CD71 (green), xCT (red), and ATF3 (yellow) in tumor tissues. Magnification: 40× and 400×. * *p* < 0.05, *** *p* < 0.001, **** *p* < 0.0001.

We also established a nude mouse xenograft model bearing ATF3-knockdown pNEN cells and found that the tumor-suppressive effect of QY21 in vivo was significantly attenuated upon ATF3 knockdown. Tumors in the ATF3-knockdown group treated with QY21 exhibited larger volumes and heavier weights compared with those in the wild-type cell group receiving QY21 treatment (Figure 8A–C). Furthermore, we detected ferroptosis-related molecules in the xenograft tumors via IHC and immunofluorescence co-staining and consistently observed that ATF3 knockdown reversed QY21-induced ferroptosis in vivo (Figure 8D–G).

Taken together, these findings suggested that ATF3 knockdown suppressed QY21-triggered ferroptosis and impaired the tumor-suppressive effect of QY21 in pNENs.

## 4. Discussion

Bruton’s tyrosine kinase has emerged as a highly promising therapeutic target in recent years. Apart from hematological malignancies and autoimmune diseases, BTKis also have attracted considerable interest in solid tumors [37,38]. This is not only because BTK modulates oncogenic pathways such as NF-κB and ERK through its expression in myeloid cells and other tumor microenvironment components, but also because ibrutinib acts as a multi-kinase inhibitor targeting epidermal growth factor receptor (EGFR), human epidermal growth factor receptor 2 (HER2), and other kinases implicated in solid tumors, thereby suppressing tumor proliferation, invasion, and angiogenesis [38]. In the field of neuroendocrine tumors, Laura Soucek et al. discovered that ibrutinib could inhibit mast cell degranulation in insulinoma transgenic mice, subsequently inducing extensive apoptosis of tumor cells and endothelial cells as well as vascular regression [34]. Regrettably, a phase II clinical trial demonstrated that single-agent ibrutinib failed to achieve objective tumor responses in patients with advanced NENs, with a median PFS of merely 3 months, indicating unsatisfactory clinical efficacy of ibrutinib against pNENs [36]. Whether alternative BTK inhibitors could serve as promising therapeutic agents for pNENs remains to be elucidated. In this study, we addressed this issue and drew the following conclusions. First, we found the novel pyrrolopyrimidine-based BTK inhibitor, QY21, exerted potent antiproliferative activity against pNENs with a lower IC_50_ in vitro and a more robust suppression of subcutaneous tumor growth in vivo than ibrutinib. Second, transcriptome sequencing analysis indicated that QY21 restrains the proliferation of pNENs by triggering ferroptosis. Third, QY21 induced ferroptosis by upregulating ATF3 expression. Finally, genetic inhibition of ATF3 reversed QY21-induced ferroptosis and abrogated its antitumor efficacy against pNENs. Collectively, our study characterized a newly synthesized BTK inhibitor QY21, as a potent anti-pNENs agent that suppresses tumor proliferation via ATF3-mediated ferroptosis.

Despite weaker predicted BTK binding affinity from molecular docking relative to ibrutinib, QY21 still exerted potent anti-proliferative activity against pNENs with a lower IC_50_ in both BON-1 and QGP-1 cells than ibrutinib (Figure 1E–H). This inconsistency may arise from intrinsic limitations of static rigid-body docking, which neglects BTK conformational dynamics, intracellular compound bioavailability, membrane permeability, and potential multi-pathway inhibitory effects of QY21 within tumor cells. In vivo xenograft models derived from BON-1 cells further confirmed that QY21 produced a more robust suppression of tumor volume and weight reduction than ibrutinib and no obvious body weight loss or no obvious organ was observed in QY21 group (Figure 3). These findings indicated QY21 as a potent anti-pNENs agent with a favorable safety profile.

Ferroptosis is an iron-dependent programmed cell death [22]. Excess iron ions generate hydroxyl radicals through the Fenton reaction, which in turn trigger membrane lipid peroxidation. Apart from direct cytotoxic effects, ferroptosis plays an important role in tumor initiation and progression through multiple mechanisms, including disrupting cellular metabolism, facilitating tumor immune responses, and modulating the tumor microenvironment [22,39]. In the field of pNENs, our previous studies have demonstrated that ferroptosis is crucial for pNEN cells death mediated by agents such as PROTAC-Surufatinib and EZH2 inhibitors [40,41]. In the present study, KEGG enrichment analysis identified ferroptosis as a core biological pathway modulated by QY21 (Figure 4C). QY21 upregulated pro-ferroptotic proteins ACSL4 and CD71 while downregulating antioxidant proteins xCT and SCD1, accompanied by excessive ROS and lipid peroxidation accumulation (Figure 5). All these findings indicated that QY21 promoted ferroptosis in pNEN cells. To further validate this conclusion, we performed rescue experiments using Fer-1 and found that Fer-1 alleviated QY21-induced ROS and lipid peroxidation accumulation and reversed the anti-proliferative effect of QY21 on pNEN cells (Figure 5). These findings suggest that ferroptosis plays an essential role in the QY21-mediated inhibition of pNENs proliferation. However, we only measured total intracellular ROS and lipid peroxidation levels, but did not further distinguish specific ROS and reactive nitrogen species (RNS) subtypes. Profiling individual ROS/RNS species in future work will help dissect the precise oxidative signaling pathways that drive QY21-mediated ferroptosis in pNEN cells.

Based on the results of transcriptome sequencing, we speculated that ATF3 may exert a vital function in QY21-induced ferroptosis in pNENs. Accumulating evidence has revealed that ATF3 serves as a key regulator of ferroptosis. On one hand, ATF3 elevates intracellular iron accumulation by upregulating transferrin receptor 1 and downregulating ferroportin. Additionally, it modulates ACSL4, SCD1 and lipoxygenase pathways to alter lipid peroxidation levels. Meanwhile, ATF3 directly suppresses GPX4 and xCT, impairing the glutathione antioxidant system, thereby facilitating ferroptosis in gastric cancer, endometrial carcinoma, hepatocellular carcinoma and other malignancies [42,43]. In the present study, we report for the first time the function of ATF3 in pNENs. We verified intrinsically low ATF3 expression in pNEN cell lines and clinical tumor tissues (Figure 4H–K). ATF3 overexpression promotes ferroptosis and restrains cell proliferation in pNENs (Figure 6). Conversely, ATF3 knockdown attenuates the inhibitory effect of QY21 on pNEN cells, accompanied by reduced lipid peroxidation and ROS accumulation (Figure 7). Consistent findings were observed in nude mouse xenograft tumor models. Collectively, these bidirectional genetic rescue experiments solidly demonstrate that QY21 accelerates ferroptosis in pNEN cells via targeting ATF3. However, the precise molecular mechanism by which ATF3 drives ferroptosis remains to be further elucidated.

However, our study still has several limitations. First, all functional validations relied solely on established pNEN cell lines, without verification in patient-derived primary cells. Our animal model relied on subcutaneous xenograft tumors in nude mice, precluding evaluation of the impact of QY21 on the full adaptive immune response within the tumor microenvironment. Future studies incorporating humanized PDX models, orthotopic models, or patient-derived organoid-immune cell co-cultures will be essential to dissect the potential immune-modulatory component of mechanism of QY21 and strengthen the translational relevance of QY21. Second, our in vivo toxicity assessment was confined to body weight monitoring and histopathological examination of major organs, without hematological and serum biochemical analyses. Future studies should focus on these issues. Systemic pharmacokinetic and pharmacodynamic experiments, as well as dose-dependent toxicity studies, are needed to strengthen the translational rationale. Third, the upstream signaling cascade linking QY21-mediated BTK inhibition to ATF3 transcriptional upregulation remains to be fully elucidated. Transcriptomic data suggest that the MAPK/ERK pathway is potentially involved, as it was significantly enriched upon QY21 treatment (Adjusted *p* value = 0.00024, Table S5). Future studies combining phospho-specific immunoblotting with ATF3 promoter-luciferase reporter assays will be required to validate whether pharmacological modulation of the MAPK/ERK pathway directly impacts QY21-induced ATF3 expression, thereby delineating the precise upstream regulatory axis.

## 5. Conclusions

In conclusion, this study demonstrates that QY21 inhibits the progression of pNENs by upregulating ATF3 to facilitate ferroptosis in pNEN cells both in vitro and in vivo. This ATF3-mediated ferroptosis pathway may provide a novel preclinical strategy for pNENs treatment. Further investigations based on patient-derived models and systematic pharmaceutical characterizations are required to validate the translational potential of QY21 for clinical application.

## Figures and Tables

**Table 1:** IC_50_ of novel pyrrolopyrimidine-based BTKis in pNEN cells.

	IC_50_ (μM)			IC_50_ (μM)	
Compound	BON-1	QGP-1	Compound	BON-1	QGP-1
QY1	32.72	34.24	QY12	23.98	89.73
QY2	703.5	-	QY13	105.62	-
QY3	49.62	65.91	QY14	54.49	67.39
QY4	59.76	73.53	QY15	135.9	-
QY5	60.42	77.32	QY16	22.41	27.32
QY6	41.93	56.74	QY17	138.2	-
QY7	87.63	-	QY18	98.76	-
QY8	33.57	55.45	QY19	88.85	-
QY9	36.21	46.13	QY20	21.88	37.49
QY10	309.14	-	QY21	8.53 ± 3.83	24.47 ± 5.61
QY11	174.84	-	QY22	23.67	29.23

## Data Availability

The data presented in this study are available on request from the corresponding author.

## Supplementary Materials

The following supporting information can be downloaded at https://www.mdpi.com/article/10.3390/cancers18142277/s1: Supplementary Materials S1: Original images of Western blotting. Table S1: Short hairpin targets. Table S2: Primers of genes. Table S3: Antibody information. Table S4: Molecular docking parameters of QY21 and ibrutinib against BTK. Table S5: Pathways significantly enriched upon QY21 treatment.

## Abbreviations

The following abbreviations are used in this manuscript:

NENs	neuroendocrine neoplasms
pNENs	pancreatic neuroendocrine neoplasms
GEP-NENs	Gastroenteropancreatic neuroendocrine neoplasms
BTK	Bruton’s tyrosine kinase
BTKi	Bruton’s tyrosine kinase inhibitor
ATF3	Activating transcription factor 3
OS	overall survival
PFS	progression-free survival
ROS	reactive oxygen species
qRT-PCR	quantitative real-time polymerase chain reaction
WB	Western blotting
DMF	dimethylformamide
PFA	paraformaldehyde
EdU	5-ethynyl-2′-deoxyuridine
BSA	bovine serum albumin
IC_50_	Half-maximal inhibitory concentration
DEGs	differentially expressed genes
IHC	immunohistochemical
ACSL4	acyl-CoA synthetase long-chain family member 4
GPX4	glutathione peroxidase 4
xCT	solute carrier family 7 member 11
SCD1	stearoyl-CoA desaturase 1
CD71	transferrin receptor 1
Fer-1	ferrostatin-1
EGFR	epidermal growth factor receptor
HER2	human epidermal growth factor receptor 2
RNS	reactive nitrogen species

## References

[1] Tacelli M., Gentiluomo M., Biamonte P., Castano J.P., Berković M.C., Cives M., Kapitanović S., Marinoni I., Marinovic S., Nikas I. (2025). Pancreatic neuroendocrine neoplasms (pNENs): Genetic and environmental biomarkers for risk of occurrence and prognosis. Semin. Cancer Biol..

[2] Das S., Dasari A. (2021). Epidemiology, Incidence, and Prevalence of Neuroendocrine Neoplasms: Are There Global Differences?. Curr. Oncol. Rep..

[3] Dasari A., Wallace K., Halperin D.M., Maxwell J., Kunz P., Singh S., Chasen B., Yao J.C. (2025). Epidemiology of Neuroendocrine Neoplasms in the US. JAMA Netw. Open.

[4] Hourcade J.P., O’ROrke M., Chrischilles E., Rudzianski N.J., Gryzlak B., Peterman K., Ortman C., Wolstencroft S., Bean M., Gellerman E. (2025). Personal Health Record Software for Neuroendocrine Tumors: Patient-Centered Design Approach. JMIR Hum. Factors.

[5] Darbà J., Marsà A. (2019). Exploring the current status of neuroendocrine tumours: A population-based analysis of epidemiology, management and use of resources. BMC Cancer.

[6] Xu Z., Wang L., Dai S., Chen M., Li F., Sun J., Luo F. (2021). Epidemiologic Trends of and Factors Associated With Overall Survival for Patients With Gastroenteropancreatic Neuroendocrine Tumors in the United States. JAMA Netw. Open.

[7] Hallet J., Meloche-Dumas L., Sarzo C., Court C., Armah J., Ding A., Chan W.C., Singh S. (2026). Incidence and Survival of Gastro-Entero-Pancreatic Neuroendocrine Neoplasms in the Contemporary Era. JAMA Netw. Open.

[8] Guo Z., Han X., Chen J., Zhang L., Sun J., Li Q., Du X., Dong F., Xiu P. (2025). Recent advances and treatment strategies in liver metastasis of pancreatic neuroendocrine tumors. Cancer Metastasis Rev..

[9] Kasai Y., Ito T., Masui T., Nagai K., Anazawa T., Uchida Y., Ishii T., Umeshita K., Eguchi S., Soejima Y. (2024). Liver transplantation for gastroenteropancreatic neuroendocrine liver metastasis: Optimal patient selection and perioperative management in the era of multimodal treatments. J. Gastroenterol..

[10] Perez K., Del Rivero J., Kennedy E.B., Basu S., Chauhan A., Connolly H.M., Dasari A.N., Gangi A., Clarke C.N., Hallet J. (2026). Symptom Management for Well-Differentiated Gastroenteropancreatic Neuroendocrine Tumors: ASCO Guideline. JCO Oncol. Pract..

[11] Vijayvergia N., Lee L.S., Katona B.W. (2026). Gastroenteropancreatic Neuroendocrine Tumors. Gastroenterology.

[12] Ito T., Masui T., Komoto I., Doi R., Osamura R.Y., Sakurai A., Ikeda M., Takano K., Igarashi H., Shimatsu A. (2021). JNETS clinical practice guidelines for gastroenteropancreatic neuroendocrine neoplasms: Diagnosis, treatment, and follow-up: A synopsis. J. Gastroenterol..

[13] Shah M.H., Goldner W.S., Benson A.B., Bergsland E., Blaszkowsky L.S., Brock P., Chan J., Das S., Dickson P.V., Fanta P. (2021). Neuroendocrine and Adrenal Tumors, Version 2.2021, NCCN Clinical Practice Guidelines in Oncology. J. Natl. Compr. Cancer Netw..

[14] Pavel M., Öberg K., Falconi M., Krenning E.P., Sundin A., Perren A., Berruti A. (2020). Gastroenteropancreatic neuroendocrine neoplasms: ESMO Clinical Practice Guidelines for diagnosis, treatment and follow-up. Ann. Oncol..

[15] Xu J., Shen L., Bai C., Wang W., Li J., Yu X., Li Z., Li E., Yuan X., Chi Y. (2020). Surufatinib in advanced pancreatic neuroendocrine tumours (SANET-p): A randomised, double-blind, placebo-controlled, phase 3 study. Lancet Oncol..

[16] Yao J.C., Pavel M., Lombard-Bohas C., Van Cutsem E., Voi M., Brandt U., He W., Chen D., Capdevila J., De Vries E.G.E. (2016). Everolimus for the Treatment of Advanced Pancreatic Neuroendocrine Tumors: Overall Survival and Circulating Biomarkers From the Randomized, Phase III RADIANT-3 Study. J. Clin. Oncol..

[17] Raymond E., Dahan L., Raoul J.-L., Bang Y.-J., Borbath I., Lombard-Bohas C., Valle J., Metrakos P., Smith D., Vinik A. (2011). Sunitinib Malate for the Treatment of Pancreatic Neuroendocrine Tumors. N. Engl. J. Med..

[18] Rinke A., Müller H.-H., Schade-Brittinger C., Klose K.-J., Barth P., Wied M., Mayer C., Aminossadati B., Pape U.-F., Bläker M. (2009). Placebo-Controlled, Double-Blind, Prospective, Randomized Study on the Effect of Octreotide LAR in the Control of Tumor Growth in Patients With Metastatic Neuroendocrine Midgut Tumors: A Report From the PROMID Study Group. J. Clin. Oncol..

[19] Rinke A., Wittenberg M., Schade-Brittinger C., Aminossadati B., Ronicke E., Gress T.M., Müller H.-H., Arnold R., for the PROMID Study Group (2017). Placebo-Controlled, Double-Blind, Prospective, Randomized Study on the Effect of Octreotide LAR in the Control of Tumor Growth in Patients with Metastatic Neuroendocrine Midgut Tumors (PROMID): Results of Long-Term Survival. Neuroendocrinology.

[20] Faivre S., Niccoli P., Castellano D., Valle J.W., Hammel P., Raoul J.-L., Vinik A., Van Cutsem E., Bang Y.-J., Lee S.-H. (2017). Sunitinib in pancreatic neuroendocrine tumors: Updated progression-free survival and final overall survival from a phase III randomized study. Ann. Oncol..

[21] Xu J., Shen L., Li J., Zhou Z., Bai C., Li Z., Chi Y., Li E., Yu X., Xu N. (2025). Surufatinib in advanced neuroendocrine tumours: Final overall survival from two randomised, double-blind, placebo-controlled phase 3 studies (SANET-ep and SANET-p). Eur. J. Cancer.

[22] Zhou Q., Meng Y., Li D., Yao L., Le J., Liu Y., Sun Y., Zeng F., Chen X., Deng G. (2024). Ferroptosis in cancer: From molecular mechanisms to therapeutic strategies. Signal Transduct. Target. Ther..

[23] Ru Q., Li Y., Chen L., Wu Y., Min J., Wang F. (2024). Iron homeostasis and ferroptosis in human diseases: Mechanisms and therapeutic prospects. Signal Transduct. Target. Ther..

[24] Kang R., Liu J., Wang J., Kroemer G., Tang D. (2026). Translating ferroptosis into oncology: Challenges, opportunities and future directions. Nat. Rev. Clin. Oncol..

[25] Ku H.-C., Cheng C.-F. (2020). Master Regulator Activating Transcription Factor 3 (ATF3) in Metabolic Homeostasis and Cancer. Front. Endocrinol..

[26] Chen M., Liu Y., Yang Y., Qiu Y., Wang Z., Li X., Zhang W. (2021). Emerging roles of activating transcription factor (ATF) family members in tumourigenesis and immunity: Implications in cancer immunotherapy. Genes Dis..

[27] Zhao X., Chen C., Qiu H., Liu J., Shao N., Guo M., Jiang Y., Zhao J., Xu L. (2025). The landscape of ATF3 in tumors: Metabolism, expression regulation, therapy approach, and open concerns. Pharmacol. Res..

[28] Tian K., Wei J., Wang R., Wei M., Hou F., Wu L. (2023). Sophoridine derivative 6j inhibits liver cancer cell proliferation via ATF3 mediated ferroptosis. Cell Death Discov..

[29] Feng Z., Cao K., Sun H., Liu X. (2024). SEH1L siliencing induces ferroptosis and suppresses hepatocellular carcinoma progression via ATF3/HMOX1/GPX4 axis. Apoptosis.

[30] Fu D., Wang C., Yu L., Yu R. (2021). Induction of ferroptosis by ATF3 elevation alleviates cisplatin resistance in gastric cancer by restraining Nrf2/Keap1/xCT signaling. Cell. Mol. Biol. Lett..

[31] Liu J., Lu X., Zeng S., Fu R., Wang X., Luo L., Huang T., Deng X., Zheng H., Ma S. (2024). ATF3-CBS signaling axis coordinates ferroptosis and tumorigenesis in colorectal cancer. Redox Biol..

[32] Wang L., Zhang Z., Yu D., Yang L., Li L., He Y., Shi J. (2023). Recent research of BTK inhibitors: Methods of structural design, pharmacological activities, manmade derivatives and structure–activity relationship. Bioorg. Chem..

[33] Fares A., Uribe C.C., Martinez D., Rehman T., Rondon C.S., Sandoval-Sus J. (2024). Bruton’s Tyrosine Kinase Inhibitors: Recent Updates. Int. J. Mol. Sci..

[34] Soucek L., Buggy J.J., Kortlever R., Adimoolam S., Monclús H.A., Allende M.T.S., Swigart L.B., Evan G.I. (2011). Modeling Pharmacological Inhibition of Mast Cell Degranulation as a Therapy for Insulinoma. Neoplasia.

[35] Sun S.H., Angell C.D., Savardekar H., Sundi D., Abood D., Benner B., DiVincenzo M.J., Duggan M., Choueiry F., Mace T. (2023). BTK inhibition potentiates anti-PD-L1 treatment in murine melanoma: Potential role for MDSC modulation in immunotherapy. Cancer Immunol. Immunother..

[36] Al-Toubah T., Schell M.J., Cives M., Zhou J.-M., Soares H.P., Strosberg J.R. (2020). A Phase II Study of Ibrutinib in Advanced Neuroendocrine Neoplasms. Neuroendocrinology.

[37] Cool A., Nong T., Montoya S., Taylor J. (2024). BTK inhibitors: Past, present, and future. Trends Pharmacol. Sci..

[38] Szklener K., Michalski A., Żak K., Piwoński M., Mańdziuk S. (2022). Ibrutinib in the Treatment of Solid Tumors: Current State of Knowledge and Future Directions. Cells.

[39] Wu Y., Li H., Yue K., Jing C., Duan Y. (2025). Ferroptosis in cancer: Metabolism, mechanisms and therapeutic prospects. Mol. Cancer.

[40] He N., Xu Y., Yan L., Hu P., Bai J., Xue B., Hu C., Lu X., Liu M., Ye M. (2026). HMGCS1 as a potential mediator of resistance to EZH2 inhibition via ferroptosis mediated by PI3K/AKT/mTOR pathway in the pancreatic neuroendocrine neoplasms. Endocr.-Relat. Cancer.

[41] Xue B., Yan L., Ye M., Gu D., Qian J., He N., Hu P., Lu F., Lu X., Liu M. (2025). PROTAC-Surufatinib Suppresses Pancreatic Neuroendocrine Neoplasms Progression by Inducing Ferroptosis through Inhibiting WNT/β-catenin Pathway Mediated by HMOX1. Int. J. Biol. Sci..

[42] Jia M., Shi M., Zhao Y., Li Y., Liu X., Zhao L. (2025). The role of ATF3 in the crosstalk between cellular stress response and ferroptosis in tumors. Crit. Rev. Oncol..

[43] Liu S., Li Z., Lan S., Hao H., Baz A.A., Yan X., Gao P., Chen S., Chu Y. (2024). The Dual Roles of Activating Transcription Factor 3 (ATF3) in Inflammation, Apoptosis, Ferroptosis, and Pathogen Infection Responses. Int. J. Mol. Sci..

