# Supplementary material for: A Novel Bruton’s Tyrosine Kinase Inhibitor Suppresses Pancreatic Neuroendocrine Neoplasms Progression via ATF3-Induced Ferroptosis
Source: Cancers (Basel). 2026 Jul 15;18(14):2277. doi: 10.3390/cancers18142277 (PMC13407139; doi:10.3390/cancers18142277)
Supplement: Supplementary file 1 [file cancers-18-02277-s001.zip › Table S1 to S5.pdf]

Table S1: Short hairpin targets.

Gene names	Target sequence (5'-3')
ATF3 sh1	GCTGAACTGAAGGCTCAGATT
ATF3 sh2	GATATACATGCTCAACCTTCA
ATF3 sh3	GAGGCGACGAGAAAGAAATAA

Table S2: Primers of genes.

Gene names	Sequence (5'-3')
ATF3 forward	CTGGAAAGTGTGAATGCTGAAC
ATF3 reverse	ATTCTGAGCCCGGACAATAC
HMOX1 forward	AAGACTGCGTTCCTGCTCAAC
HMOX1 reverse	AAAGCCCTACAGCAACTGTCG
AKR1C1 forward	GGCTTTGTTAGGCAACTGTGT
AKR1C1 reverse	AAGGCAGCGAAGGATTCAGA
GAPDH forward	GGAGCGAGATCCCTCCAAAAT
GAPDH reverse	GGCTGTTGTCATACTTCTCATGG
BTK forward	GCTCAAAAACGTAATCCGGTACA
BTK reverse	GTCTTCCGGTGAGAACTCCC

Table S3: Antibody information.

Antibody	Company	Catalogue
GAPDH	Proteintech	60004-1-Ig
ATF3	Affinity	DF3110
BTK	Proteintech	21581-1-AP
GPX4	Abmart	T56959
SCD1	Proteintech	28678-1-AP
xCT	Abmart	T57046
CD71	Proteintech	10084-2-AP
ACSL4	Proteintech	22401-1-AP

Table S4: Molecular docking parameters of QY21 and ibrutinib against BTK.

Compound	Ibrutinib	QY21
**Binding energy (kcal/mol)**	−11.27	−9.57
**Inhibition constant (μM)**	0.005	0.097
**Final Intermolecular Energy (kcal/mol)**	−11.7	−9.60

Table S5: Pathways significantly enriched upon QY21 treatment.

Pathway_Name	Rich.Factor	*p*.Value	Sig.Sign.P	Q.value	Sig.Sign.Q	ZScore
Circadian rhythm	0.2059	2.44E-05	****	0.006381	**	4.973459
MAPK signaling pathway	0.0623	0.000237	***	0.030932	*	4.798026
Alcoholism	0.0657	0.002307	**	0.156137	ns	−0.14991
Colorectal cancer	0.0889	0.002571	**	0.156137	ns	3.88198
Mitophagy - animal	0.0789	0.003195	**	0.156137	ns	5.346797
Amphetamine addiction	0.0933	0.003589	**	0.156137	ns	−0.635
Axon guidance	0.0635	0.004403	**	0.164185	ns	2.923174
Parathyroid hormone synthesis, secretion and action	0.0698	0.007181	**	0.204022	ns	1.353431
Growth hormone synthesis, secretion and action	0.0687	0.007917	**	0.204022	ns	1.585258
Autophagy - animal	0.0611	0.008298	**	0.204022	ns	7.385337

* $p < 0.05$, ** $p < 0.01$, *** $p < 0.001$, **** $p < 0.0001$; ns, not significant.
