# Supplementary figures and images for: A Novel Bruton’s Tyrosine Kinase Inhibitor Suppresses Pancreatic Neuroendocrine Neoplasms Progression via ATF3-Induced Ferroptosis
Source: Cancers (Basel). 2026 Jul 15;18(14):2277. doi: 10.3390/cancers18142277 (PMC13407139; doi:10.3390/cancers18142277)

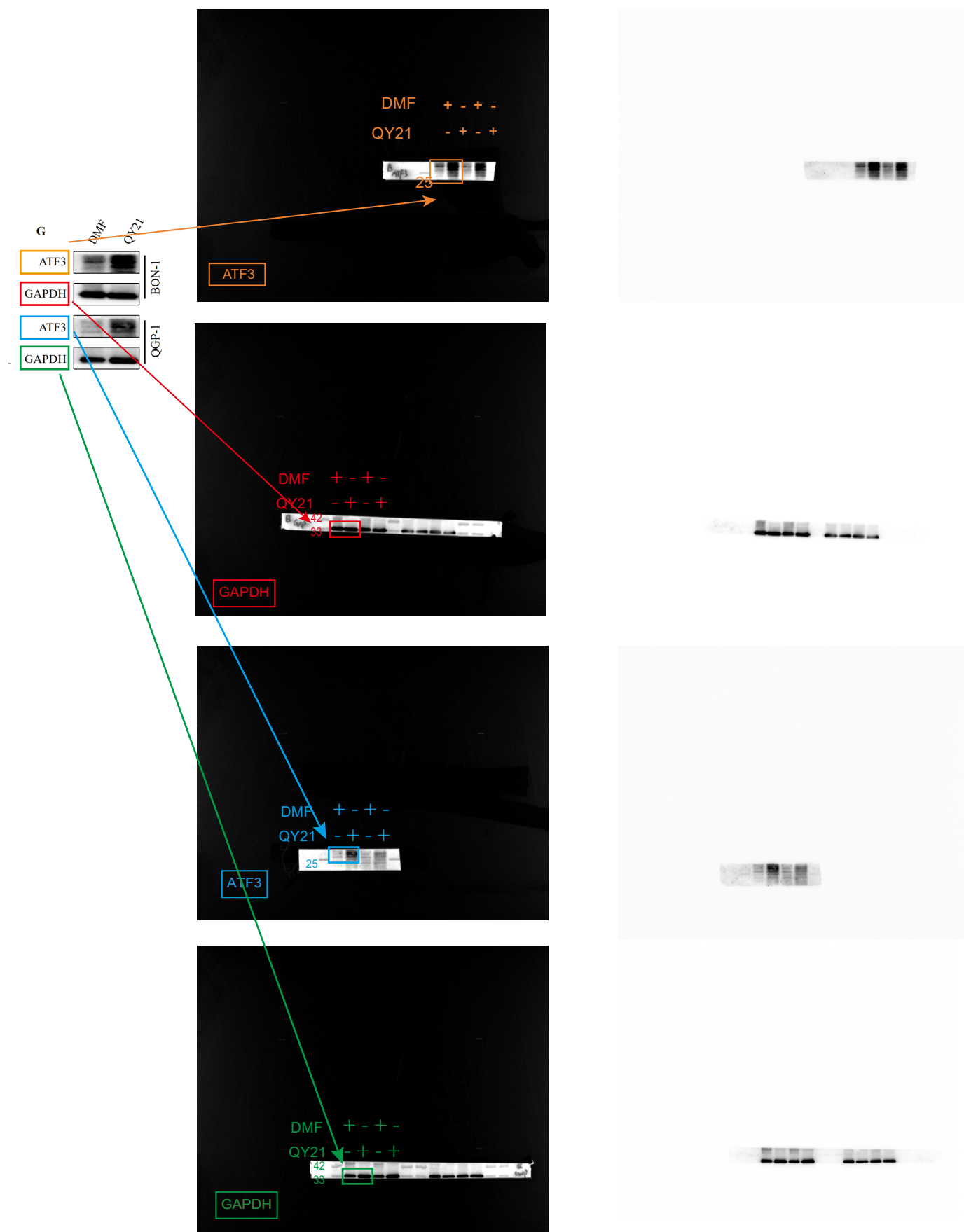

Figure S1 Original images of western blots for Figure 4G

Supplement: Supplementary file 1 [file cancers-18-02277-s001.zip › Figure S1-4G.pdf]

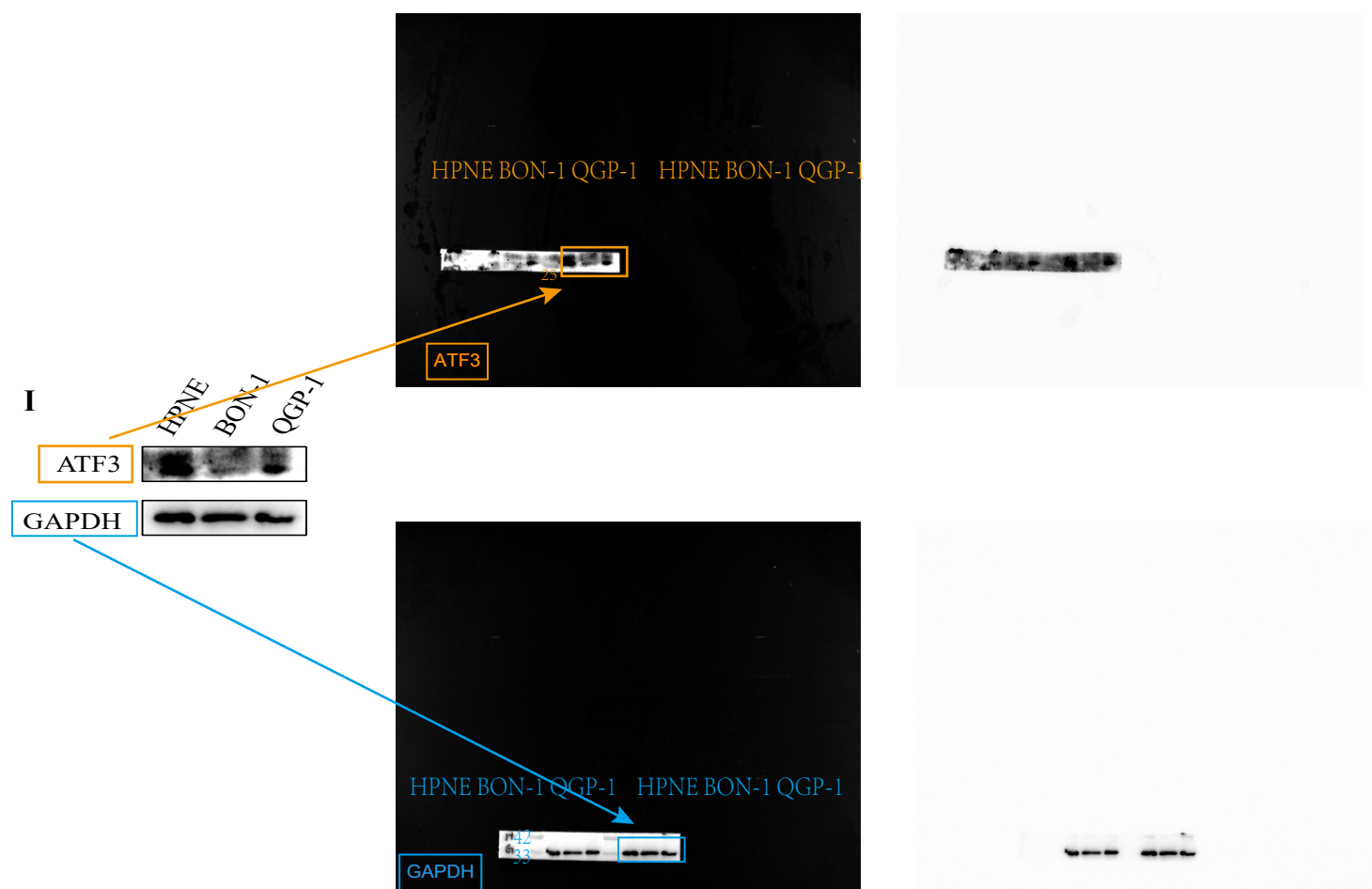

Figure S2 Original images of western blots for Figure 4I

Supplement: Supplementary file 1 [file cancers-18-02277-s001.zip › Figure S2-4I.pdf]

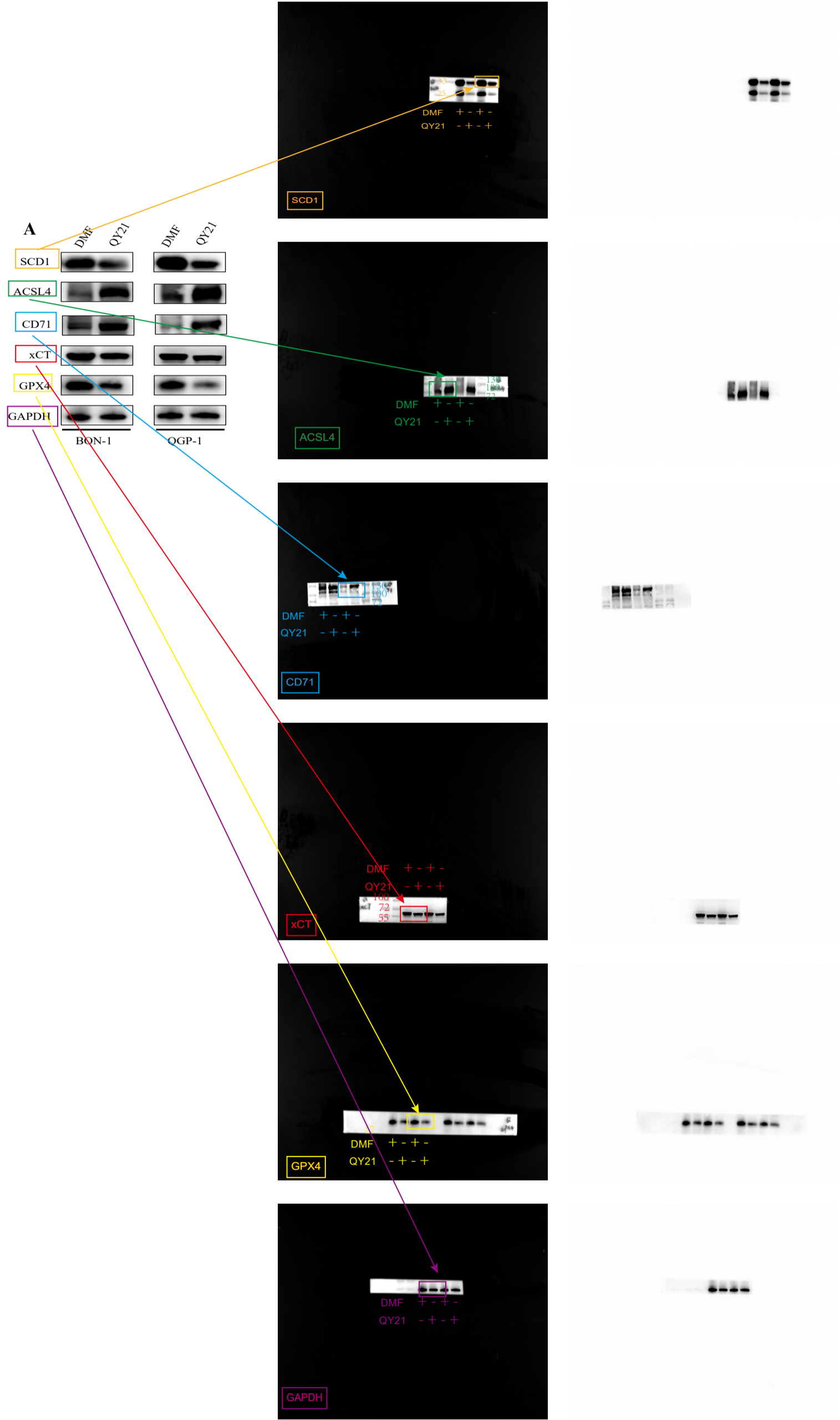

Figure S4 Original images of western blots for Figure 5A (Right panel)

Supplement: Supplementary file 1 [file cancers-18-02277-s001.zip › Figure S4 (Right panel).pdf]

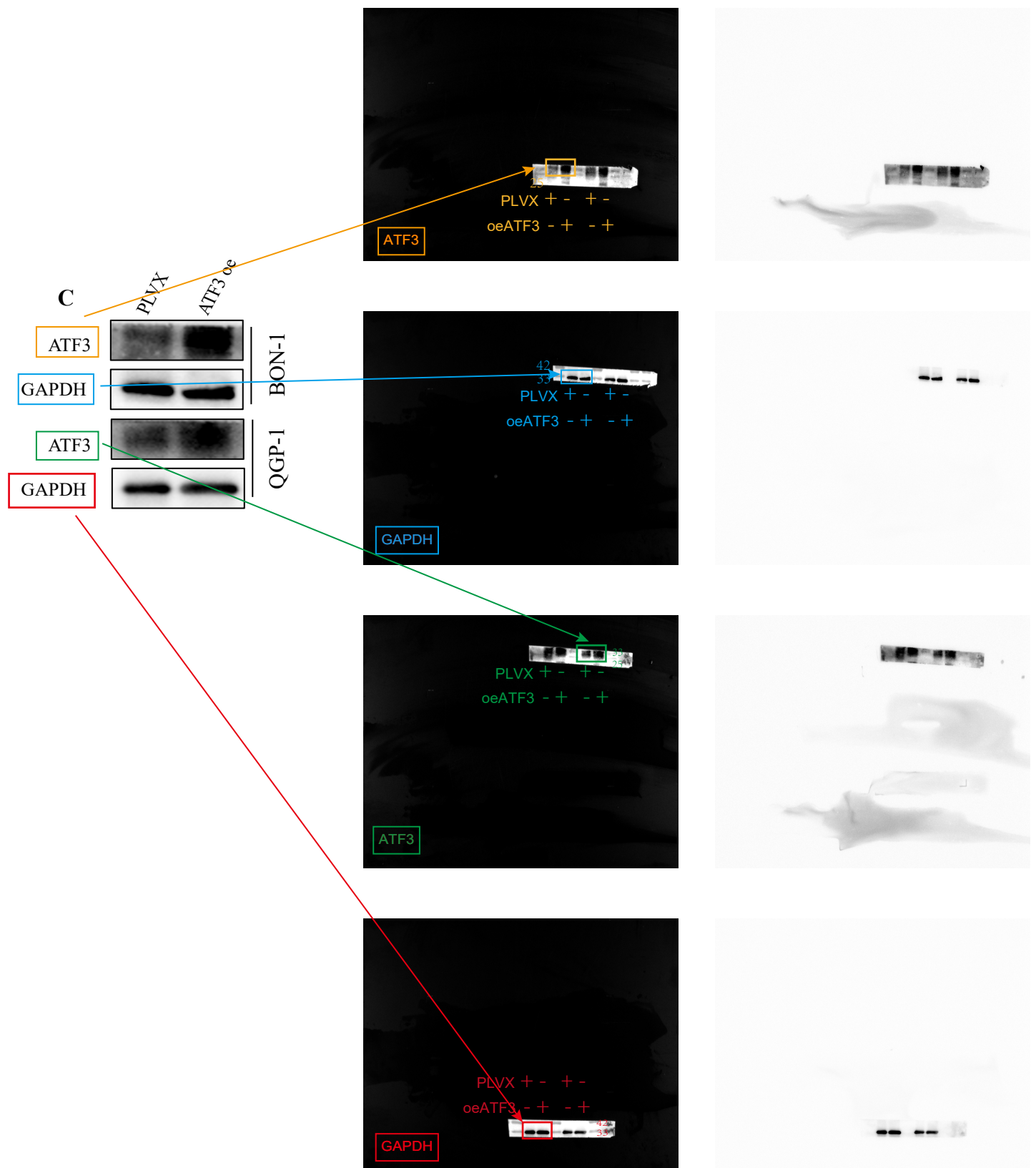

Figure S5 Original images of western blots for Figure 6C

Supplement: Supplementary file 1 [file cancers-18-02277-s001.zip › Figure S5-6C.pdf]

P

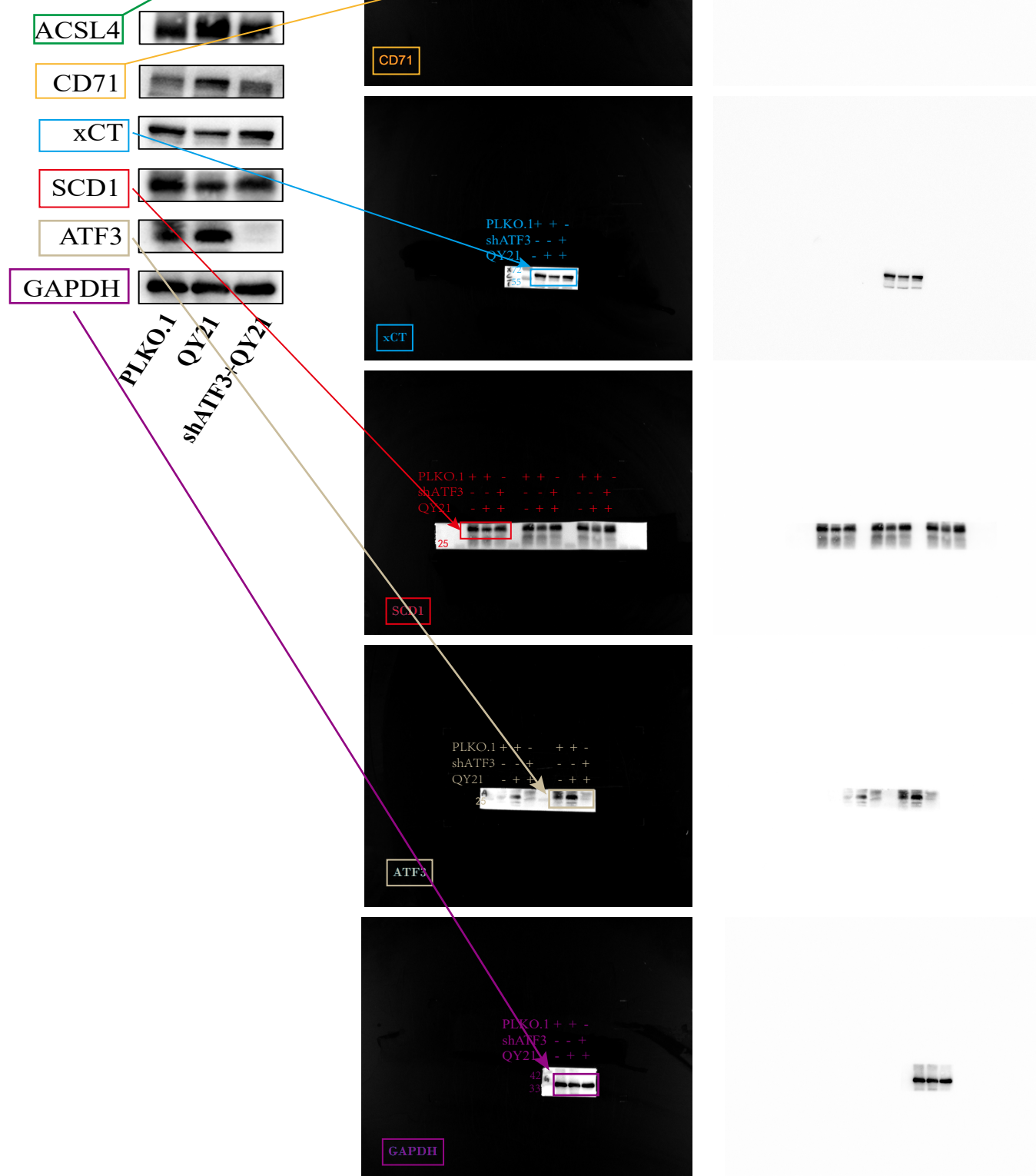

Figure S7 Original images of western blots for Figure 7P

Supplement: Supplementary file 1 [file cancers-18-02277-s001.zip › Figure S7-7P.pdf]
