# Supplementary material for: A Novel Bruton’s Tyrosine Kinase Inhibitor Suppresses Pancreatic Neuroendocrine Neoplasms Progression via ATF3-Induced Ferroptosis
Source: Cancers (Basel). 2026 Jul 15;18(14):2277. doi: 10.3390/cancers18142277 (PMC13407139; doi:10.3390/cancers18142277)
Supplement: Supplementary file 1 [file Figure S6-7C.pdf › Figure S6-7C.pdf]

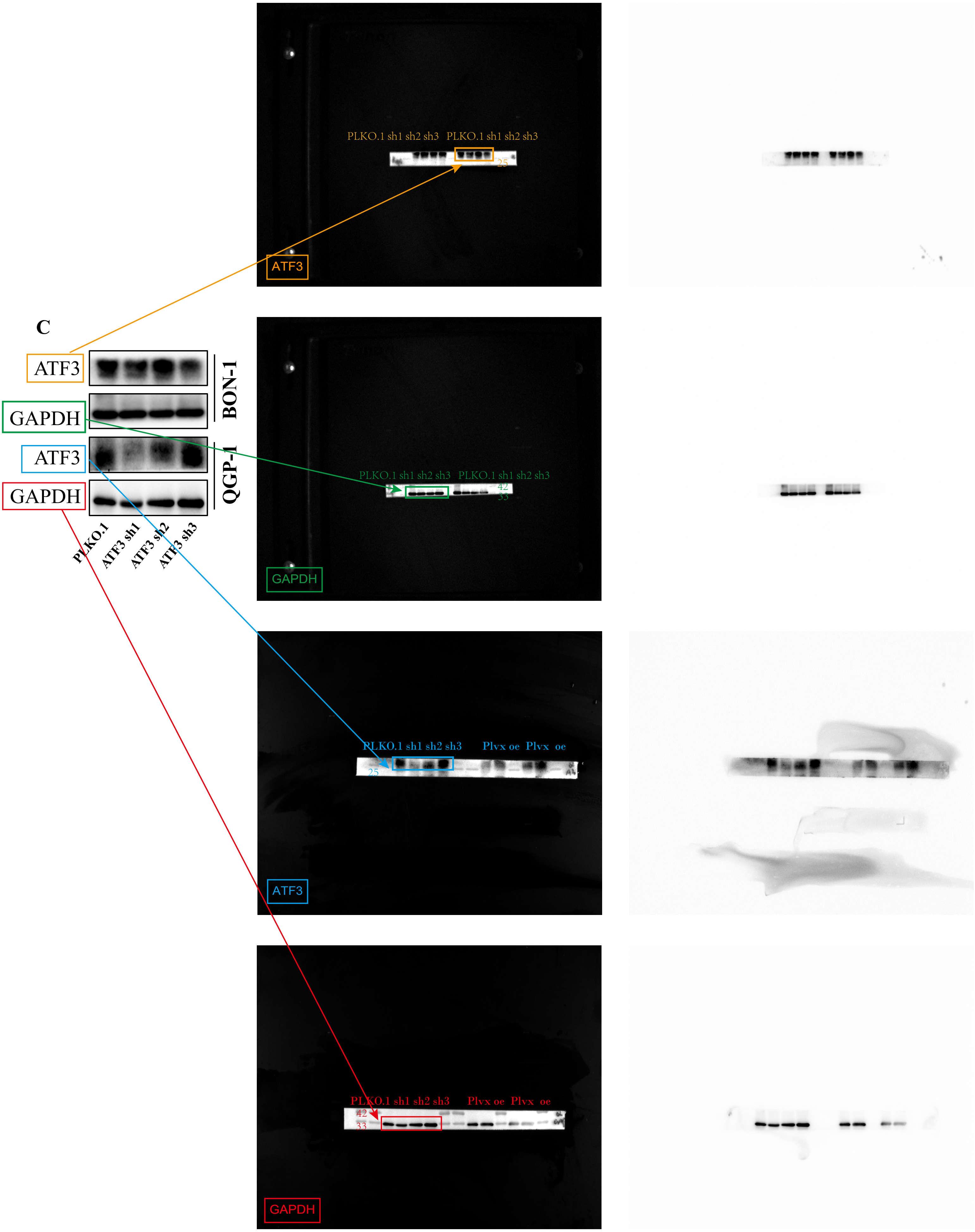

Figure S6 Original images of western blots for Figure 7C
